# Toroidal qubits: naturally-decoupled quiet artificial atoms

**DOI:** 10.1038/srep16934

**Published:** 2015-11-26

**Authors:** Alexandre M. Zagoskin, Arkadi Chipouline, Evgeni Il’ichev, J. Robert Johansson, Franco Nori

**Affiliations:** 1 Physics Department, Loughborough University, Loughborough LE11 3TU, United Kingdom; 2 iTHES Research Group, RIKEN, Saitama 351-0198, Japan; 3 Theoretical Physics and Quantum Technologies Department, Moscow Institute for Steel and Alloys, 119049 Moscow, Russia; 4 Technische Universität Darmstadt, Institut für Mikrowellentechnik und Photonik, Merckstr. 25, 64283 Darmstadt, Germany; 5 Leibniz Institute of Photonic Technology, P.O. Box 100239, D-07702 Jena, Germany; 6 Center for Emergent Matter Science, RIKEN, Saitama 351-0198, Japan; 7 Physics Department, The University of Michigan, Ann Arbor, Michigan, 48109-1040, USA

## Abstract

The requirements of quantum computations impose high demands on the level of qubit protection from perturbations; in particular, from those produced by the environment. Here we propose a superconducting flux qubit design that is naturally protected from ambient noise. This decoupling is due to the qubit interacting with the electromagnetic field only through its toroidal moment, which provides an unusual qubit-field interaction, which is suppressed at low frequencies.

A key requirement for quantum computing hardware is a low enough decoherence rate, which would allow either the implementation of quantum error correction schemes^1^,^2^, or the operation of an adiabatic optimization process^3^. Despite significant recent improvements in their performance^4^, superconducting qubits are still more vulnerable to decoherence produced by local (i.e., by the qubit itself) and ambient (originating from the environment, including the control and readout circuitry) noise, than some other platforms, such as spin- and ion trap-based ones^5^,^6^,^7^,^8^. Nevertheless, scalability and well-developed fabrication techniques make superconductor-based implementations a very attractive option, both for adiabatic^9^,^10^ and circuit-based^11^,^12^ quantum computing. Various designs of “quiet” or “silent” superconducting qubits have been proposed (e.g.^13^,^14^), but they involve exotic superconductors and do not protect against the intrinsic low frequency noise. The latter, especially the ubiquitous 1/f-noise present both in the qubits and control and readout circuitry^15^,^16^,^17^, poses a serious challenge to coherent operation of qubit arrays.

Here we investigate a qubit design that is naturally insensitive to low-frequency noise and is well protected from other ambient noise sources, and therefore could be a good candidate for a superconducting qubit. This qubit is also interesting from the point of view of investigating interesting and largely unexplored phenomena on the interaction of an electromagnetic field with toroidal multipoles in the quantum regime. (Encoding qubits in higher conventional multipoles was investigated for charge qubits by Storcz *et al.*^18^).

This paper is organized as follows: First, we give a brief review of toroidal multipole moments. Then we introduce two qubit designs that are based on superconducting Josephson-junction circuits with toroidal geometries and analyze the qubit-field interaction and the corresponding coupling strengths. Further, we discuss possible decoherence processes and rates for the toroidal qubit designs summarize the obtained results.

The textbook multipole expansion of the electromagnetic field of a system of charges and currents routinely neglects a series of terms, which first appear in the higher orders of the expansion and that are independent of electric and magnetic multipoles. The toroidal multipoles were predicted by Zel’dovich in 1957^19^; there he gave an example of the lowest-order (dipolar) toroidal moment, which corresponds to the fields of a toroidal solenoid in the limit when its size tends to zero (Fig. 1a). The external magnetic field of such a structure is zero, but its interaction with an applied external magnetic field **H** is nonzero and proportional to 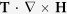, where **T** is the toroidal dipole moment (see below). It is part of the third-order expansion of the charge/current densities, so it appears in the usual expansions together with the octupole and magnetic quadrupole moments^20^. The toroidal moments are nontrivial objects, which have been considered mainly in nuclear physics, but did not attract too much attention in electrodynamics. Nevertheless, in the past years, numerous researchers are studying toroidal structures in optics and radio-frequencies^21^,^22^,^23^,^24^; the toroidal ordering was observed in natural crystals (for a recent review see, e.g., Ref. 25).

Toroidal structures have interesting properties including: (1) *absence of generated fields*, for zero frequency, and the same for nonzero-frequency using anapoles, e.g. a combination of a toroid and a dipole; (2) *violation of the reciprocity theorem*^26^; and (3) the anapole is also a potential candidate for dark matter in the universe^27^. Here we study the concept of quantum toroidal structures and show a direct application of these for quantum information processing.

Let us briefly recapitulate the properties of a dipolar toroidal moment (see, e.g.^28^). Consider the toroidal current distribution of Fig. 1a. The sheet current density **J** produces the magnetization **M** inside the torus:


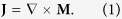


Since 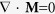, then


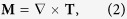


where **T** is the dipolar toroidal moment. At large distances from the torus (i.e., in the limit when the diameter of the tube, and then the radius of the torus are taken to zero) the toroidal moment of the system remains finite and characterizes its electromagnetic potentials.

Remarkably, a toroidal moment couples to the *time derivatives* of the external electromagnetic field. In particular, for a toroidal dipole in the limit of slow spatial variation of the external field, the coupling potential is^28^


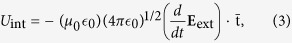


where 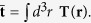 For Fig. 1, with the torus axis directed along **n**, diameter 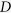, crossection 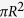, and considering the toroidal dipole as a toroidal solenoid with 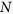 turns and current 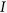 in each turn, we find^28^,^29^


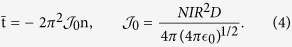


Therefore, the coupling between the toroidal dipole and the electromagnetic field is given by


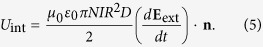


The sign of this coupling is positive (i.e., if 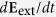 is co-directional with 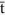, then the energy of the system increases). This can be seen directly from the fourth Maxwell’s equation: The additional magnetic field 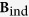 inside the torus, induced by the growing electric flux, will add to the field 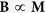 (see Fig. 1b). Since the coupling to the external electric field is proportional to its time derivative, a dipole toroidal moment is *insensitive to low-frequency electric noise*.

## Results

### Toroidal qubit

We consider two possible designs of toroidal qubits (see Fig. 2). It can be seen from their lumped-elements circuit that these designs are topologically identical to one of the first successful superconducting qubit designs: The persistent current flux qubit^30^, i.e., a superconducting loop of negligible self-inductance interrupted by three (or more) Josephson junctions. Its further variations, e.g the 8-shaped (gradiometric) qubit^31^, or a qubit with an additional loop with trapped fluxoid used as a phase bias tool^32^, allowed to improve the performance staying within a two-dimensional design.

In our proposed device, instead of a flat loop, the equilibrium currents flow in three dimensions, along a completely, or partially, closed toroidal surface formed by the superconducting layers and tunneling barriers. In the “closed” version, the superconducting layers completely enclose the internal volume, where the magnetic field generated by the Josephson currents is confined. The advantage of this design (the toroidal current flow and thus zero leakage of the magnetic field to the outside) is counterbalanced by the impossibility to bias the qubit to the vicinity of the degeneracy point by the external magnetic flux. Therefore, it is necessary to use a 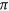-junction, and to fine-tune the qubit by the external bias current, as in the case of a phase qubit^33^. While qubits with 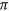-junctions have been successfully demonstrated, their fabrication and incorporation in more complex designs remain a challenge^34^.

The “open” version, which is similar to a classical implementation of a toroidal moment^21^, only approximates the toroidal current, but due to the holes in the electrode B, it can be tuned by the external magnetic field and does not require a 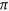-junction. It has a gradiometric design, making it *less sensitive* to the ambient noise^39^ and to some extent compensates for the deviation from the “ideal” toroidal design.

The Lagrangian of the system, as a function of 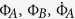, and 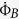, is given by (see Ref. 35, Ch.2):


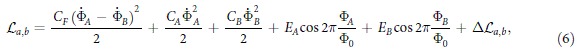


where


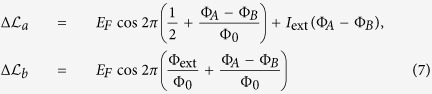


describe the potential (Josephson) energy. The sub-indices 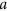 and 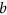 refer to the toroidal qubits shown in 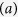 and 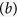, respectively, in Fig. 2. The variables


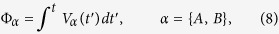


are related to the voltages at the corresponding nodes, measured with respect to the ground node (which can be chosen arbitrarily), and 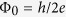 is the magnetic flux quantum. Finally, *I*_ext_ and 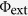 are the external tuning parameters (bias current and the magnetic flux through the corresponding loop, respectively).

To decouple the time derivatives in the Lagrangian, we introduce new variables


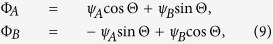



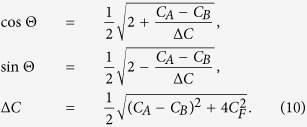


Then the Lagrangian becomes


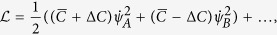


where 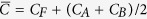. We can introduce the canonical momenta (“generalized electric charges”)


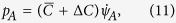



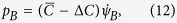


and the Hamiltonian then becomes


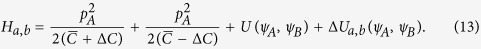


The potential energy terms in Eq. (13) are:


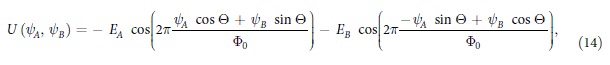



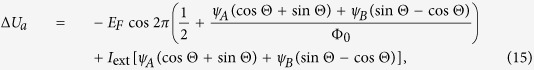



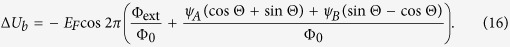


The electric charge on a given node equals


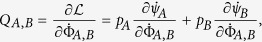


that is,


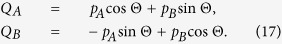


**Figure 1:**
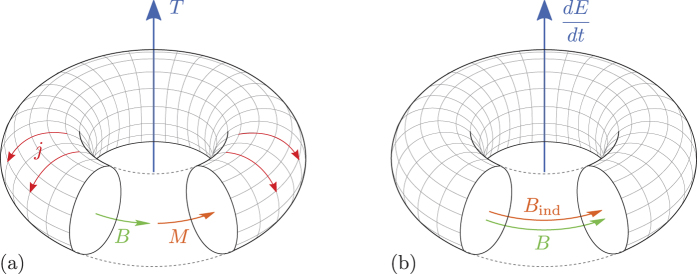
(**a**) Current 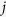, magnetic field 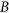, magnetic moment 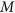 and dipolar toroidal moment 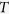 of a toroidal coil. (**b**) Toroidal coil interacting with an external electric field.

This allows us to estimate the electric dipole moment of the qubit. In the case of a realistic choice of parameters (see below) this moment is negligibly small. This is to be expected, since the original persistent current qubit was specifically designed to minimize the influence of the electric charge noise^30^. The toroidal moment of the qubit is determined by the Josephson current flow pattern through the coefficient 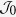 in Eq. (4), which now becomes proportional to the current operator. We can approximately express it as


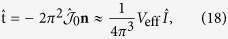


where 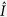 is the operator of the Josephson current flowing between the electrodes A and B (that is, circulating around the qubit loop—see the equivalent scheme), and *V*_eff_ is the effective volume encased by the current (in the case of a torus, Fig. 1, 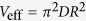).

### Qubit-field coupling strength

From the above considerations, the coupling between the qubit and the external electric field is given by


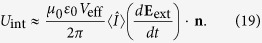


Estimating 
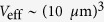
 and 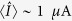, we see that


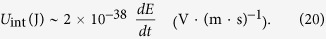


**Figure 2:**
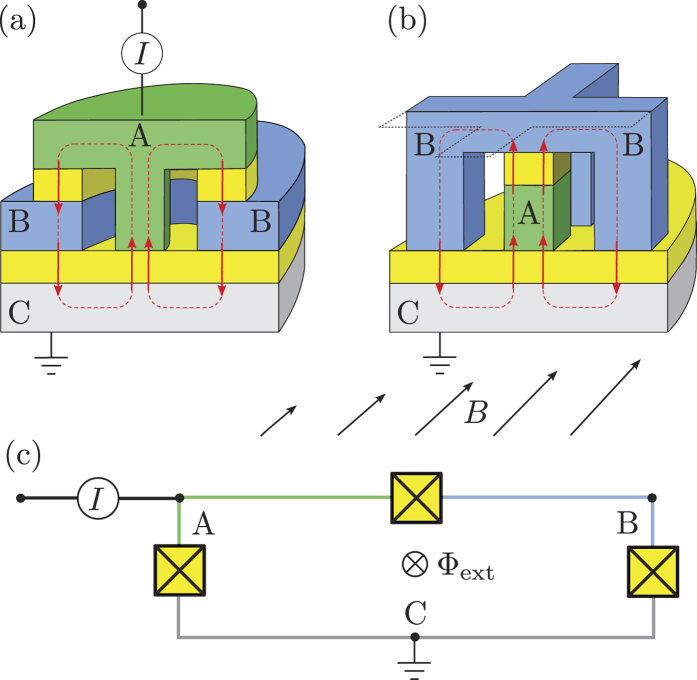
“Cutout” diagrams of toroidal qubits: (**a**) “Closed” version; (**b**) “Open” version. The superconducting electrodes (A—green, B—blue, and C—grey) are separated by tunneling barriers (yellow). One of the two possible directions of the circulating Josephson currents is shown. (**c**) Equivalent circuit.

For a field with amplitude 100 kV/m and frequency 10 GHz this yields the interaction strength ~1.5 × 10^−24^ J, or ~2 GHz. The toroidal moment of the qubit depends on its quantum state. In the “physical” basis of states 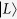 and 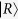 (i.e., those with the Josephson currents flowing like in Fig. 2b or in the opposite direction) it can be written as


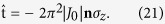


**Figure 3:**
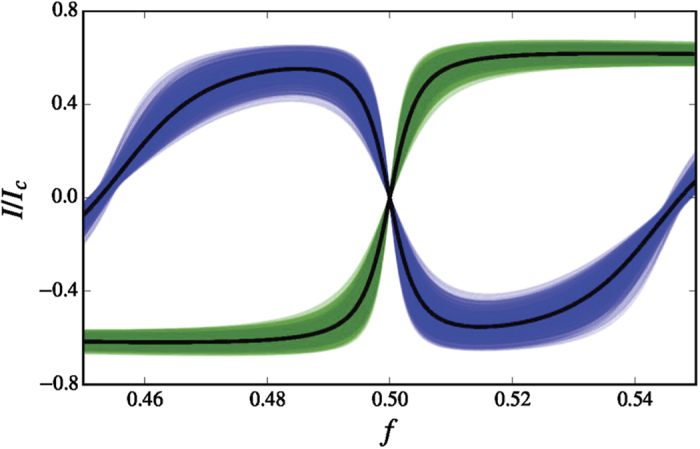
The circulating current *I* for the ground state (green) and the first excited state (blue) of the flux-biased toroidal qubit, versus the reduced magnetic flux *f* = Φ_*ext*_/Φ_0_. Here we use the parameters 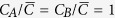, 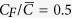, 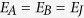, 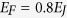, and 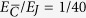, where 
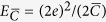
 is the average charging energy and 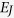 the Josephson energy corresponding to the critical current 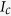. Typical values of the critical current and charging energy considered here are 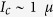
A and 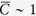 fF. The thin curves represent the critical current 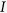 for 1000 random realization that include a 10% disorder in 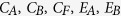 and 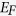. We note that the qubit is stable with respect to moderate variations in these parameters.

The Josephson currents for the two lowest-energy states of the external-flux-biased qubit design, as a function of the reduced magnetic flux *f*, is shown in Fig. 3.

The effective qubit Hamiltonian can be obtained in a standard way by quantizing Eq. (13) and considering only the subspace spanned by the two lowest-lying states^30^:


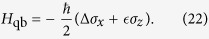


The bias 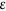 is controlled by the parameters 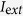 and 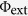, while the tunneling splitting Δ is determined by the ratio of charging and Josephson energies of the junctions (see Refs 30,35) and is typically in the GHz range.

If the electric field, with which the qubit interacts, is parallel to the *z* axis, then the field-qubit interaction term in the Hamiltonian is


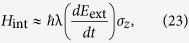


where


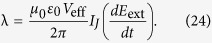


### Discussion

Decoherence in Josephson-junction-based superconducting qubits can stem from a variety of sources^38^,^40^. In particular, fluctuations in the electromagnetic field—due to charge fluctuations in the qubit’s surrounding—can couple to the charge degree of freedom of the superconducting islands in the qubit, leading to both relaxation and dephasing. Usually such charge noise couples to the qubit through a dipole interaction, and is often the leading source of decoherence in charge-qubit designs. Magnetic flux noise can be another major source of decoherence in Josephson-junction based qubits, and this noise can couple to the qubit through its circuit loops. Flux noise is the leading source of decoherence in flux qubits, especially when operated away from the optimal working point^42^. At the optimal working point both charge and flux qubits decouple, to first order, from the charge and flux noise. Also, decoherence due to charge noise is mitigated in the transmon qubit design, which is almost completely insensitivity to charge noise due to its suppressed charge dispersion^41^. However, higher-order processes, e.g., fluctuations from the optimal point, still results in significant decoherence and sets practical limits for current qubit designs. In addition to charge and flux noise, superconducting qubits are sensitive to critical current fluctuations, quasiparticle effects, dielectric losses, strongly coupled localized defects, and other noise sources^15^,^16^.

The closed toroidal qubit is protected from the ambient low-frequency noise (e.g., 1/f-noise). Its reaction to high-frequency ambient noise is less important, since it is routinely filtered out in standard experimental setups. The open toroidal qubit is also well protected, partly due to its gradiometric design. As we have shown, the numerical calculations with reasonable parameters also show tolerance of the system to the parameters dispersion. Insensitivity to ambient noise is especially important from the point of view of scalability, reducing the effects of stray fields in a multiqubit structure with numerous control wires. Note also that experimental investigation of a “classical” RF SQUID of toroidal design^43^ demonstrated its negligible sensitivity to ambient fields. The decoherence from intrinsic noises, such as produced by the surface sources^15^, cannot be suppressed this way and remains a major source of decoherence, but it can be significantly reduced by the optimization of the fabrication processes.

Despite looking exotic, toroidal qubits can be fabricated using current modern superconducting niobium or aluminum technology^36^,^37^. For example, the structure (a) of Fig. 2 is formed from the stack containing two Josephson junction in series. By making use of electron beam lithography and etching, the inner dimension of the torus and the outer dimensions of the junction can be shaped simultaneously. Depositing dielectric and lift-off consequently will result in a qubit of type (a) without the upper electrode. After a planarization of the structure this electrode can be deposited on top, thus completing the fabrication process. Fabrication of the control and readout circuitry will not present any challenges beyond the routine planar fabrication.

### Conclusions

Due to its effective decoupling from the environment, only the decoherence sources inside the toroidal section of the qubit limit the qubit decoherence time. The material properties of the dielectric inside the torus thus acquire the key importance. This presents both a challenge and an opportunity. We know that a drastic increase of decoherence can be achieved by improving the quality of the tunneling barrier in a qubit^38^. However, the relative insensitivity of the toroidal design to the external noise would make it a good tool for the investigation of low-temperature noise properties of different dielectrics for microwave quantum engineering.

## Additional Information

**How to cite this article**: Zagoskin, A. M. *et al.* Toroidal qubits: naturally-decoupled quiet artificial atoms. *Sci. Rep.* **5**, 16934; doi: 10.1038/srep16934 (2015).
